# Visualization of Painful Experiences Believed to Trigger the Activation of Affective and Emotional Brain Regions in Subjects with Low Back Pain

**DOI:** 10.1371/journal.pone.0026681

**Published:** 2011-11-02

**Authors:** Kazuhiro Shimo, Takefumi Ueno, Jarred Younger, Makoto Nishihara, Shinsuke Inoue, Tatsunori Ikemoto, Shinichirou Taniguchi, Takahiro Ushida

**Affiliations:** 1 Multidisciplinary Pain Center, Aichi Medical University, Aichi, Japan; 2 Department of Neuropsychiatry, Kyushu University, Fukuoka, Japan; 3 Department of Anesthesia, School of Medicine, Stanford University, Stanford, California, United States of America; 4 NPO Pain Medicine Research Information Center, Kochi, Japan; 5 Department of Orthopaedic Surgery, Kochi Medical School, Kochi, Japan; Tokyo Metropolitan Institute of Medical Science, Japan

## Abstract

In the management of clinical low back pain (LBP), actual damage to lower back areas such as muscles, intervertebral discs etc. are normally targeted for therapy. However, LBP may involve not only sensory pain, but also underlying affective pain which may also play an important role overall in painful events. Therefore we hypothesized that visualization of a painful event may trigger painful memories, thus provoking the affective dimension of pain. The present study investigated neural correlates of affect processing in subjects with LBP (n = 11) and subjects without LBP (n = 11) through the use of virtual LBP stimuli. Whole brain functional magnetic resonance imaging (MRI) was performed for all subjects while they were shown a picture of a man carrying luggage in a half-crouching position. All subjects with LBP reported experiencing discomfort and 7 LBP subjects reported experiencing pain. In contrast to subjects without LBP, subjects with LBP displayed activation of the cortical area related to pain and emotions: the insula, supplementary motor area, premotor area, thalamus, pulvinar, posterior cingulate cortex, hippocampus, fusiform, gyrus, and cerebellum. These results suggest that the virtual LBP stimuli caused memory retrieval of unpleasant experiences and therefore may be associated with prolonged chronic LBP conditions.

## Introduction

Psychological factors are known to affect the subjective experience of pain. Pain catastrophizing is one such maladaptive response to pain that is characterized by heightened pain intensity [1], increased disability [2]and difficulty disengaging from pain [3]. Recently, functional neuroimaging techniques have been developed that allow the neural correlates of psychological states to be explored. The blood oxygenation level-dependent contrast (BOLD-fMRI) is currently the most popular tool for mapping human brain activity [4]. Pain-related brain activations which could be considered as psychological factors have been reported in various studies. In healthy volunteers, several brain regions, including the primary and secondary somatosensory cortices, insula, anterior cingulate cortex (ACC), thalamus, and motor cortex, respond to real noxious stimuli and are regarded as part of the “pain matrix” [5], [6]. However, it is also known that the expectation of pain can evoke brain activation patterns resembling that of a real pain experience [7].

In a previous study [8], [9], Ogino reported that the imagination of pain even without physical injury engages the cortical representations of the pain-related neural network. Also, we reported that prior pain experiences can strongly affect pain anticipation and associated brain activations. We have also found that the anticipation of painful stimuli can cause the activation of cortical areas underlying pain-related affect in chronic neuropathic pain patients [10]. Activation in the brain during the visualization of a painful experience was found in the ACC and the medial prefrontalcortex (MPFC), which are regions known to be areas associated with pain and affect processing. Similar activations were found to be correlated with pain catastrophizing in individuals with fibromyalgia [11]. In that study, pain catastrophizing was associated with greater activity in the dorsolateral prefrontal cortex, rostral ACC, and MPFC, regions implicated in pain vigilance, attention and awareness [12], [13], [14], [15]. These results suggest that pain-related neuronal activities might reflect the development and maintenance of chronic pain syndromes.

Low back pain (LBP) is one of the most common chronic pain syndromes. A recent fMRI study in humans reported actual LBP-related cerebral substrates [16]. Abnormal activations were identified in the prefrontal cortex, insula, thalamus, posterior cingulate cortex (PCC), supplementary motor area (SMA), and premotor areas (PMA) – predominantly in the right hemisphere.

We hypothesized that visualization of a painful experience would provoke unpleasant emotions, and these emotions might have a role in the maintenance of chronic pain syndromes. The present study investigated neural correlates of affect processing in subjects with nonspecific LBP and subjects without LBP by using virtual visual stimuli.

## Results

### Self-reported discomfort and pain (Table 1)

**Table 1:** Evaluations of task-related discomfort and pain.

	LBP group (n = 11)	non-LBP group(n = 11)
Experiences evoked by tasks		
Discomfort (range)	3.5 (1–6)	0
Pain (range)	2.1 (0–6)	0
RDQ (mean ± SD)	3.1±3.1	0
ODI (mean ± SD)	19.8±7.8%	0

RDQ, Roland-Morris Disability Questionnaire; ODI, Oswestry Disability Index 2.0.

All subjects in the LBP group reported discomfort associated with viewing the simulated back pain (mean NRS score, 3.5; range, 1–6). 7 of the 11 subjects in the LBP group described pain associated with the task. However, no subjects in the non-LBP group reported any discomfort or pain resulting from viewing the picture of back pain.

### fMRI results

**Figure 1:**
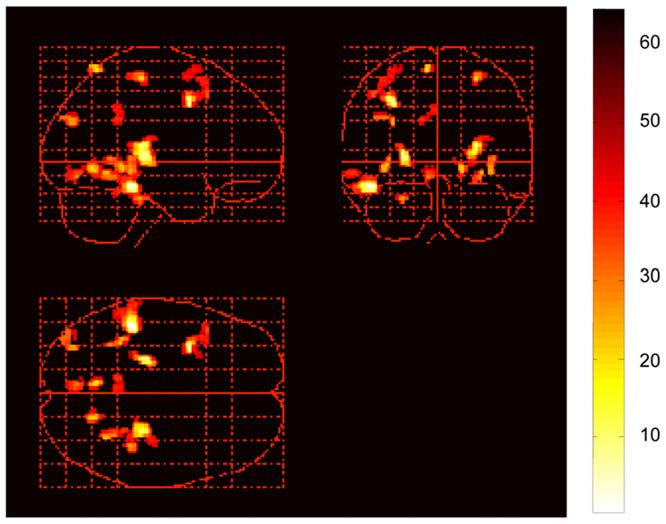
Areas of cortical activation in the LBP group compared with the non-LBP group in response to virtual LBP stimuli (task – control condition) detected by fMRI(p<0.0005, Z score>3.4, uncorrected threshold).

Compared with the non-LBP group, the LBP group demonstrated significantly more activation in the left fusiform, as well as left inferior temporal gyrus, bilateral precentral gyrus, left middle frontal gyrus, left superior frontal gyrus, bilateral thalamus, bilateral caudate, right insula, left postcentral gyrus, bilateral lingual gyrus, bilateral parahippocampal gyrus, right superior temporal gyrus, left angular gyrus, left superior occipital gyrus, left precuneus, left middle temporal gyrus, left posterior cingulate cortex (PCC), and left cerebellum (Table 2, Fig. 1). The reverse contrast showed that the LBP group had lower activations than the non-LBP group in a single cluster in right caudate (Table 2).

**Table 2:** Talairach coordinates and Broadmann's areas for regions of statistically significant activation (p<0.0005 at voxel level uncorrected threshold) in response to virtual LBP stimulation (task – control condition).

Anatomical region	Side	Coordinate	Broadmann area	Z score
LBP group as compared to non-LBP group				
Fusiform gyrus	Lt	−46, −34, −13	Area 20	4.53
Inferior temporal gyrus	Lt	−57, −43, −15	Area 37	3.60
Precentral gyrus	Lt	−32, 8, 38	Area 9	4.38
	Rt	28, −24, 56	Area 4	4.03
Middle frontal gyrus	Lt	−46, 20, 43	Area 8	3.68
		−32, 11, 60	Area 6	3.50
Superior frontal gyrus	Lt	−40, 16, 53	Area 8	3.56
Thalamus	Lt	−24, −25, 7	-	4.34
	Rt	24, −27, 0	-	3.40
Caudate	Lt	−28, −32, 13	-	3.57
	Rt	38, −35, −3	-	3.91
Insula	Rt	28, −27, 12	Area 13	4.30
	Rt	34, −20, 18	Area 13	3.50
Postcentral gyrus	Lt	−8, −55, 64	Area 7	4.07
Lingual gyrus	Rt	18, −62, 0	Area 19	3.99
	Lt	−6, −72, −5	Area 18	3.81
Parahippocampal gyrus	Lt	−36, −43, 0	Area 19	3.96
	Rt	32, −53, −4	Area 19	3.91
	Rt	28, −41, −10	Area 36	3.62
Superior temporal gyrus	Rt	40, −35, 4	Area 41	3.78
Angular gyrus	Lt	−32, −74, 30	Area 39	3.88
Superior occipital gyrus	Lt	−38, −80, 33	Area 19	3.78
Precuneus	Lt	−42, −72, 35	Area 19	3.42
Middle temporal gyrus	Lt	−60, −35, −5	Area 21	3.62
Posterior cingulate gyrus	Lt	−10, −41, 30	Area 31	3.61
	Lt	−4, −43, 37	Area 31	3.55
Cerebellum	Lt	−24, −30, −20	-	3.88
non-LBP group as compared to LBP group				
Caudate	Rt	22, −34, 20	-	3.61

**Figure 2:**
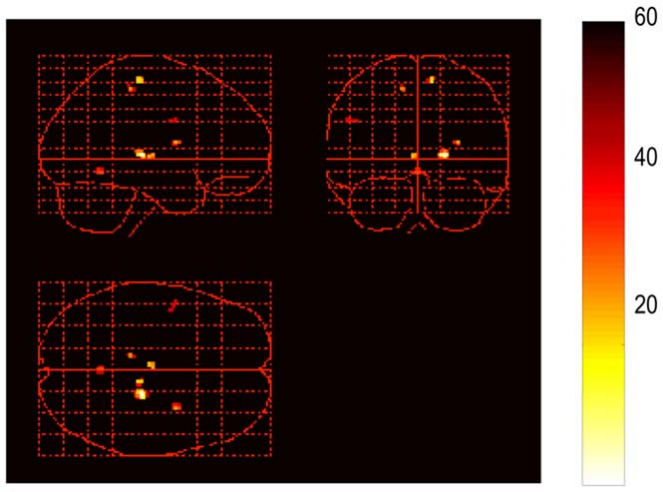
Areas of cortical activation showing an association with perceived discomfort.

**Figure 3:**
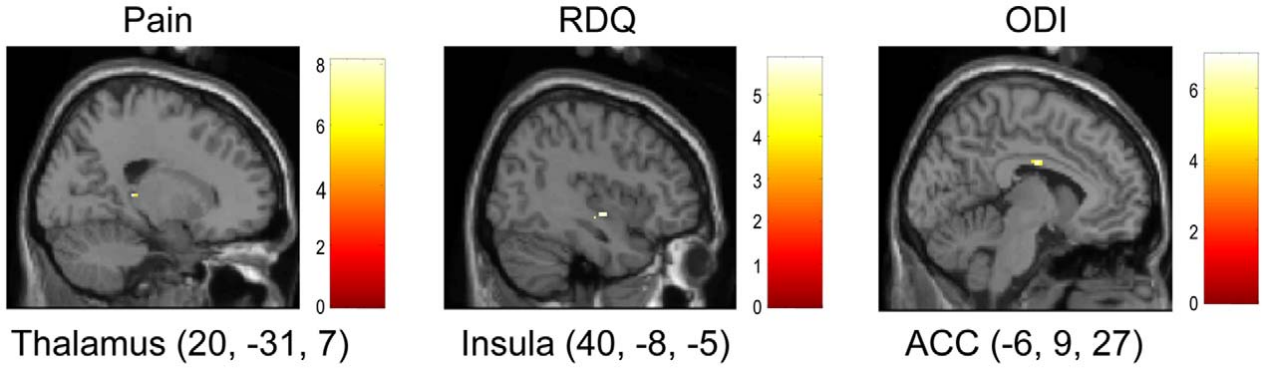
Sagittal sections showing cortical clusters where activity was linearly correlated with perceived pain, RDQ scores and ODI scores.

In the LBP group, activations related to discomfort were found in the bilateral thalamus, bilateral medial frontal gyrus, right claustrum, left cerebellum (Table 3, Fig. 2). Activations associated with self-reported pain were found in the right thalamus and right lingual gyrus. RDQ scores were associated with activation in the left ACC, and ODI scores were associated with activations in the right insula (Table 3, Fig. 3).

**Table 3:** Cortial areas showing a linear signal increase with the discomfort rating, pain rating, RDQ scores and ODI scores.

Anatomical region	Side	Coordinate	Broadmann area	Z score
Discomfort				
Thalamus	Rt	20, −23, 5	-	4.19
	Lt	−4, −17, 3	-	3.78
Medial frontal gyrus	Rt	10, −22, 58	Area 6	3.85
	Lt	−12, −28, 53	Area 6	3.70
	Lt	−50, 1, 28	Area 6	3.38
Claustrum	Rt	30, 3, 13	-	3.75
Cerebellum	Lt	0, −53, −6	-	3.57
Pain				
Thalamus	Rt	20, −31, 7	-	4.27
Lingual gyrus	Rt	8, −86, −11	Area 18	3.62
RDQ				
Anterior cingulate gyrus	Lt	−6, 9, 27	Area 24	3.99
ODI				
Insula	Rt	40, −8, −5	Area 13	3.67

RDQ, Roland-Morris Disability Questionaire; ODI, Oswestry Disability Index 2.0.

## Discussion

Our results demonstrate that viewing images of simulated back pain evoke unpleasant feelings, and specific brain activations in individuals with LBP. According to the International Association for the Study of Pain, pain is defined as, “an unpleasant sensory and emotional experience associated with actual or potential tissue damage, or described in terms of such damage”. As this definition suggests, both real pain stimuli and virtual pain experiences such as the visual stimuli in our study may play an important role in pain recognition and interpretation in the brain.

Functional MRI results showed that many of the areas described as being part of the “pain matrix” are also active during virtual pain. These results suggest that previous experiences of low back pain can sensitize an individual to pain anticipation. Activation in the insular cortex is associated with pain discrimination [17], [18], [19]. Additionally, the posterior insular cortex also plays a role in directing appropriate motor behaviors [20]. Furthermore, the insular cortex has projections to the SMA [21], [22]. The SMA and PMA are commonly activated by pain [19], [23], and usually associated with motor preparation. Activation in those areas might be associated with preparation for protective behavior against pain. In addition, we found virtual LBP stimuli led to increased activation in cerebellum. Activity in the cerebellum is frequently found in pain neuroimaging studies. Cerebellar activation is considered to be primarily associated with motor responses [13]. The need for temporally precise information may also be relevant for brain areas involved in initiating, propagating, and executing defensive motor responses to noxious stimuli [11], [13], [24], [25].

The thalamus and the pulvinar are heavily interconnected with the visual and parietal cortices. Neuroimaging studies suggest responses in the pulvinar have a spatiotopic organization that are modulated by visual attention [26], [27], [28]. These results suggest that low back pain experiences may make individuals pay more attention to pain-related visual stimuli.

Many reports identify a role of the PCC in negative emotion [29], [30], [31], [32], [33], [34], visuospatial orientation, and assessment of self-relevant sensation [35]. Exaggerated cerebral activation by pain stimuli may also be associated with pathologic pain states such as allodynia [36], [37]. Together with its possible role in inflammatory pain [38], PCC activation could possibly reflect the negative emotion and the pathologic state of pain.

We found other regions with heightened activity in LBP participants, in areas outside of the classic pain matrix. Those regions included the hippocampus, fusiform gyrus and angular gyrus. While not typically considered a nociceptive processing region, activation in the hippocampus has been previously reported to be activated in response to painful heat [14], [39] and laser stimulation [40]. The hippocampus has been traditionally associated with recent memory consolidation [41], spatial memory [42], and fear-initiated avoidance behavior [43]. The hippocampus might also play a role in memorizing the pain stimulation and preparing fear-initiated avoidance. The fusiform gyrus is often associated with facial recognition [44]. It is conceivable, therefore, that our visual stimuli (which included a human face) may have been responsible for observed activations in the fusiform gyrus. However, our visual stimuli included a human facewithout any faical expression. This might suggest that the fusiform gyrus plays another important role in the cognitive neuroscience field. The angular gyrus is associated with empathy and ‘theory of mind’ [45]. Visual stimuli may cause subjects in the LBP group to imagine self pain or feel empathy towards the individual in pain in the picture.

Via parametric analyses in the LBP group, we identified several regional activations that were associated with discomfort rating, pain rating, RDQ scores and ODI scores. The SMA and PMA were related to the discomfort rating. As indicated previously, the SMA and PMA are involved in motor preparation. Activation in those areas might therefore be associated with preparation of protective behaviors against discomfort and pain. Thalamic activation was associated with both discomfort and pain ratings. Greater insula activation was associated with higher ODI scores. The thalamus and insula are considered part of the sensory component of pain processing [46]. But, a recent study suggests that imagining oneself in painful situations is sufficient to trigger some pain sensory regions [47]. The ACC was associated with RDQ scores. The ACC is an important part of affective pain processing [48], [49] and can be activated in tasks of pain empathy [47], [50], [51], [52], [53], [54], [55]. It is unknown, therefore, whether the ACC activations, which were observed in the LBP group, were due to imagined self pain, or empathetic pain for the individual in the picture.

In this study, we showed that pain-related visual stimuli can activate several regions of the pain matrix in LBP patients, but not normal volunteers. Moreover, the pain questionnaire scores in the LBP patients were associated with greater activation of pain-processing brain regions. Functional MRI and the virtual visual tasks are non-invasive methods for probing pain-related fear and catastrophizing. These results might be applied to the evaluation of chronic pain syndromes, such as low back pain, in the future.

## Materials and Methods

We recruited subjects with nonspecific LBP (LBP group) (n = 11, 6 male, 5 female, mean age 20.4 years) and subjects without LBP (non-LBP group) (n = 11, 5 male, 6 female, mean age 21.5 years). All participants were right-handed, had no history of cerebrovascular disease, and were free from any medication within 24 hours of the study. Scores for the Roland-Morris Disability Questionnaire (RDQ) and Oswestry Disability Index 2.0 (ODI) were obtained for all participants. Participants in the LBP group reported low back pain, and a RDQ or ODI score greater than zero. Participants in the non-LBP group had never experienced low back pain lasting longer than 1 week, and their RDQ and ODI scores were zero. No participants in either group displayed any evidence of structural abnormality in the lumbar spine on MRI, or any neurologic symptoms. None reported having a history of psychiatric disorders, or currently using any psychoactive medications.

**Figure 4:**
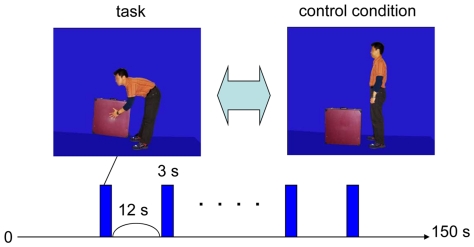
Experimental design. Subjects enrolled in the experiment were shown a picture demonstrating a man holding luggage in a half-crouching position (task picture) and a picture demonstrating a man standing in front of luggage, providing the baseline stimulation (control condition picture).

We used virtual LBP stimuli depicting a man who is carrying luggage in a half-crouching position (Fig. 4). This picture represents an action that would likely cause pain in an individual with low back pain, and may therefore cause pain anticipation in the LBP group. Participants were also shown a picture depicting a man standing in front of luggage, providing the baseline stimulation (control condition) (Fig. 4). Participants in the LBP group had painful experiences in the half-crouching posture but did not have any pain in the standing posture. In addition, the participants in the LBP group currently feel little pain in daily life. During the fMRI session, trials were presented in a fixed block design. The distance between the participants' eyes and the screen was 12.5 cm, with a visual angle of 7.4×11.3°. The trials were applied eight times in each series, with each trial presentation lasting 3 seconds. The entire functional experiment lasted 150 seconds (see details of the experimental paradigm in Fig. 4). Self-reported discomfort and pain measures were collected using a numerical rating scale after the experimental session.

Images of the entire brain were acquired using GE SIGNA 3.0 Tesla scanner. Blood oxygenation level-dependent (BOLD) signals were collected with a T2-weighted, multi-slice, gradient echo-planar imaging (EPI) sequence (TE = 35 ms, TR = 3000 ms, flip angle = 90°, slice width = 4 mm, gap = 0 mm, 36 axial slices). Participants were scanned in the supine position, with the head fixed to minimize movement artifact. During the experiment, participants were simply instructed to observe the picture on screen.

The study was approved by the Ethical Committee of Kochi Medical School. All participants were informed of the study purpose beforehand and provided written consent to participate.

Results were analyzed on a Unix workstation using SPM2 (Statistical Parametric Mapping) software; Wellcome Department of Cognitive Neurology, Institute of Neurology, London: http://www.fil.ion.ucl.ac.uk/spm). The acquired images were realigned, spatially normalized to a standard EPI template and finally smoothed with an isotropic Gaussian kernel of 6 mm FWHM (full width at half maximum). Significance was assessed using the box car approach, convolved with the canonical hemodynamic response function. Activation maps represent t-test contrasts between the different experimental conditions. To identify the neural substrates for the virtual pain task, we contrasted the task condition and control condition in the LBP and non-LBP groups. Thresholds for activation were set at p<0.0005 for the voxel level of activation, and were further corrected for multiple comparisons at the cluster extent threshold of p<0.05. The Talairach atlas was used to anatomically localize foci of significant activation [56]. Brain activation between the LBP group and the non-LBP group was statistically compared to identify the neural processing specific to the LBP group (p<0.05, corrected, one-way ANOVA).

For the LBP group only, parametric analyses were also performed to determine associations between brain activity and perceived discomfort, perceived pain, RDQ score and ODI score. Normalized ratings were introduced at the subject level, taking into account only trials from the LBP group.
